# Avian diversity patterns across residential neighbourhoods of different development stages: Insights from a subtropical waterfront metropolis in China

**DOI:** 10.3897/BDJ.13.e171169

**Published:** 2025-11-05

**Authors:** Ding Yinghong, Wu Yongshu, Pan Deng, Huang Yaling, Ran Chengyu, Li Junyi, Wang Juan, Wang Yuhan, Yang Jin, Zhu Zhipeng, Xu Xiaoling

**Affiliations:** 1 College of Landscape Architecture and Art, Fujian Agriculture and Forestry University, Fuzhou 350002, China College of Landscape Architecture and Art, Fujian Agriculture and Forestry University Fuzhou 350002 China; 2 Fujian Bird Watching Society, Fuzhou 350002, China Fujian Bird Watching Society Fuzhou 350002 China; 3 College of Architecture and Urban Planning, Tongji University, Shanghai 200092, China College of Architecture and Urban Planning, Tongji University Shanghai 200092 China; 4 College of Architecture and Planning, Fujian University of Technology, Fuzhou 350108, China College of Architecture and Planning, Fujian University of Technology Fuzhou 350108 China

**Keywords:** Urban bird diversity, Urban ecology, Residential neighbourhoods, Development stages, Urbanisation

## Abstract

This study examined bird diversity across 33 residential neighbourhoods in Fuzhou, China, representing different development stages. We found that mid-aged neighbourhoods supported the highest bird diversity and abundance, driven primarily by tree diversity and vegetation evenness, while high building density constrained habitat quality. These findings highlight the critical role of residential development history and vegetation structure in shaping urban avian communities and provide practical guidance for biodiversity-orientated urban planning.

## Introduction

With the accelerating pace of urbanisation, land-use changes and intensified human activity have profoundly reshaped urban ecosystems, posing unprecedented threats to biodiversity (Wu et al. 2004, Ives et al. 2016, Garfinkel et al. 2024). Birds, due to their high mobility, sensitivity to environmental change and ease of monitoring, are widely regarded as key indicators of urban ecological health, while also fulfilling important ecological functions such as pest control and seed dispersal (Furness and Greenwood 2013, Stephens et al. 2016). Understanding how urban environments affect bird communities has thus both theoretical and practical importance (Shochat et al. 2010).

Residential green spaces, although smaller and more fragmented than parks or wetlands, are embedded throughout the urban fabric and form a crucial part of green infrastructure (Mansor and Said 2008). From an island biogeography perspective (MacArthur and Wilson 2001), they function as “ecological islands”, where patch size, vegetation structure and connectivity influence species persistence and immigration (Goddard et al. 2010, Aronson et al. 2017, Hui et al. 2023). Previous studies confirm that well-managed residential green spaces can provide essential habitats and corridors for birds, mitigating fragmentation effects and enhancing biodiversity (Daniels and Kirkpatrick 2006, Aronson et al. 2014, Buron et al. 2022).

Built environment characteristics also strongly shape bird diversity. Residential density, building height and noise negatively affect richness and abundance, while vegetation heterogeneity promotes them (Lancaster and Rees 1979, Rodrigues et al. 2018, Xie et al. 2020, Dong et al. 2025). Yet most existing studies emphasise static spatial attributes, paying less attention to the temporal dimension of residential development. In rapidly urbanising contexts, neighbourhoods built in different periods often differ systematically in built form and vegetation structure, which, in turn, affect habitat quality and bird communities (Ran et al. 2024). Evidence from North America and elsewhere suggests that older neighbourhoods tend to support higher diversity due to more stable and heterogeneous habitats, whereas new developments often experience ecological instability linked to construction disturbance (Vale and Vale 1976, Edgar and Kershaw 1994, Pidgeon et al. 2014).

Recent studies have examined residential neighbourhoods, mostly through case-based approaches. Xie et al. (2020) found that landscape configuration, tree species richness and canopy cover strongly predicted bird species richness and abundance, with Eurasian Tree Sparrow dominating (Xie et al. 2020). Dong et al. (2025) reported that shrub cover and building characteristics significantly influenced avian diversity (Dong et al. 2025). While both provide valuable evidence, they remain geographically specific and do not address how neighbourhood development history (construction age and temporal change) shapes bird–habitat relationships — a temporal dimension considered in the present study.

Against this research background, this study focuses on 33 representative residential areas within a 1-km buffer zone along the Min River in Fuzhou. These neighbourhoods were classified into three categories, based on their construction periods. By integrating one year of field-bird observations with environmental variables of each neighbourhood, the study addresses the following key questions: (1) Do residential areas built in different periods exhibit significant differences in bird community characteristics? (2) What are the key environmental variables influencing the bird Shannon index, species richness index, Pielou evenness index and abundance? (3) Do these key factors exhibit heterogeneous effects across residential areas of different construction periods?

## Methods

### Study area and sites

Fuzhou （26°09'N~26°00'N, 119°24'E~119°42'E）is located on the south-eastern edge of the Eurasian continent, bordering the Pacific Ocean to the east and falls within a subtropical monsoon climate zone (Fuzhou Government Portal; https://www.fuzhou.gov.cn/szf/). The study area is situated along the urban section of the Min River in Fuzhou. With the accelerating pace of urbanisation, this region has developed extensive residential zones. However, little research has been conducted on how these developments affect bird communities in the adjacent residential areas along the river.

**Figure 1.**
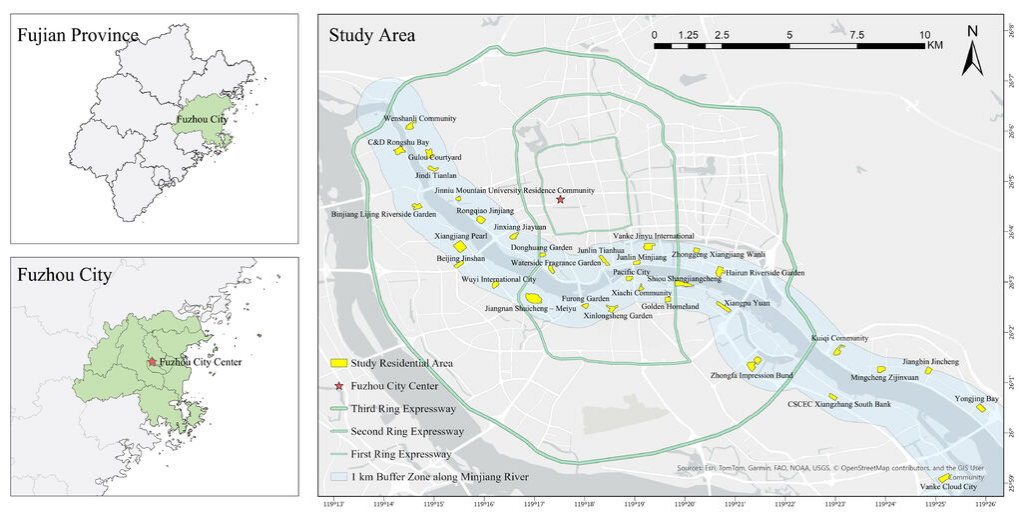
Location map of the 33 residential areas along the Min River.

In this study, we selected a section of the lower Min River near the urban core as the focal area. The study boundary was defined as a 1 km buffer zone on both sides of the river's centre-line, within which survey sites were established (Fig. 1) (Pennington and Blair 2011, Litteral and Shochat 2017, Liu et al. 2022). Following the minimum patch size standard of 2 ha specified *in Technical Guidelines for the Construction and Restoration of Urban Green Space Bird Habitats* (https://yllhj.beijing.gov.cn/sdlh/ylkj/bzgf/201911/P020220208599812707167.pdf) and based on remote sensing interpretation and field survey results, we randomly selected 33 typical residential areas within the study boundary — each with an area no less than 2 ha — as the research sites.

### Classification and spatial distribution of residential area types

In this study, the 33 selected residential areas were categorised into three types based on their year of construction: those built before 2005 (Y1), those built between 2006 and 2015 (Y2) and those built between 2016 and 2025 (Y3). Amongst them, there are 12 Y1-type residential areas, 11 Y2-type areas and 10 Y3-type areas.

**Figure 2.**
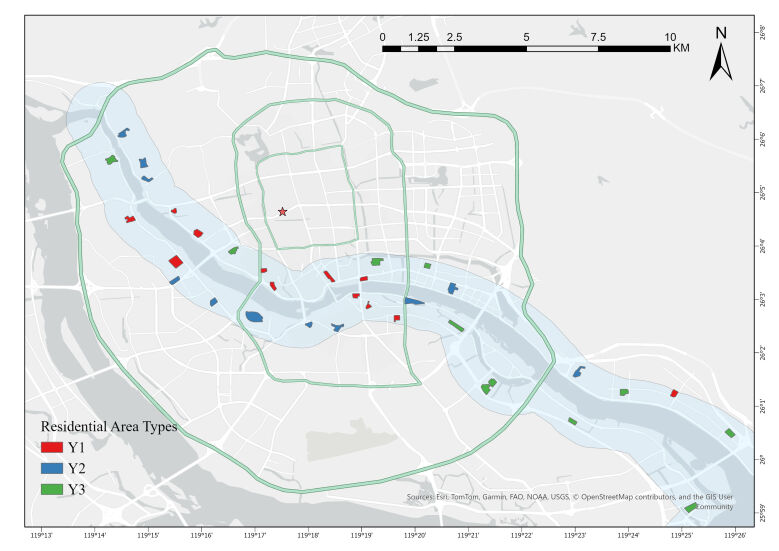
Distribution of three types of residential areas along the Min River.

The spatial distribution of the three residential types along the Min River in Fuzhou shows distinct variation (Fig. 2). The names and construction years of all residential areas are provided (Suppl. material 1).

### Data collection methods

A nested sampling design was applied to integrate bird and vegetation surveys (Yang et al. 2015). Each 100 m transect segment served as the bird survey unit, with a 20 × 20 m vegetation plot at its centre. Plot shapes were adjusted when necessary to maintain area consistency.

#### Bird survey methods

Birds were surveyed using the line-transect method. One 100 × 50 m transect was set per 2 ha of green space (Bibby 2000, Huang et al. 2022). Surveys were conducted monthly from October 2023 to September 2024 during peak morning activity (06:00 h–10:00 h) under calm, clear weather by a trained observer using 8 × 42 binoculars. Each transect was surveyed 12 times at 30-day intervals. All individuals detected within 25 m on either side were recorded. To avoid double counts, residential areas were spaced ≥ 200 m apart (Humphrey et al. 2023).

#### Vegetation survey methods

Vegetation was surveyed in spring 2024 using the five-point method. Each 20 × 20 m plot contained five 2 × 2 m shrub and one 1 × 1 m herbaceous subplot (Jiang et al. 2021, H. et al. 2022). All plant species were inventoried. For trees (> 3 m), DBH, height, crown width, abundance and height to the first branch were recorded (Academy of Inventory and Planning, S.F.A.D. 2009). Shrubs and herbaceous plants were recorded for species, height, abundance and cover.

#### Built environment data collection

Residential boundaries were obtained via the Baidu API and processed in ArcGIS Pro. Built-up area was calculated with raster analysis. Building footprints and floor-level data came from the Urban Data Analyst Platform (http://udu.org.cn/?cate=22); construction year from the Fuzhou 58 Anjuke website (https://fz.anjuke.com/). Floor area ratio, building density and average building height were derived from these data.

### Data processing methods

The environmental variables selected in this study were guided by established ecological theories and empirical findings. High building density, floor area ratio and tall structures often reduce bird diversity by intensifying noise, disturbance and habitat fragmentation (Yu et al. 2025). In contrast, tree diversity and evenness enhance habitat heterogeneity, providing varied food resources and nesting niches that support species co-existence (Batáry and Fronczek 2014). Shrub and herbaceous layers can supply additional cover and foraging substrates, though excessive density may reduce accessibility and foraging efficiency (Schlossberg et al. 2010). Together, these factors provide a comprehensive framework for assessing how vegetation and built environments shape avian communities in residential areas.

#### Plant and bird α-Diversity

To comprehensively assess the diversity of plant and bird communities, this study used R version 4.4.2 and the “vegan” package to calculate several commonly applied α-diversity indices, including the Shannon Index, species richness, Pielou’s evenness and abundance (Żmihorski et al. 2016). For plant communities, Shannon diversity, species richness and Pielou’s evenness were computed, while all four indices — Shannon diversity (BH′), species richness (BS), Pielou’s evenness (BJ′) and abundance (BA) — were calculated for bird communities. Prior to computing bird diversity indices, species accumulation curves were generated using the “iNEXT” package to evaluate sampling adequacy and predict how species richness may increase with additional sampling effort (Hsieh et al. 2016).

#### Non-parametric testing and diversity comparison

To examine differences in bird community diversity across residential areas of different construction periods, the Mann–Whitney U test was employed for non-parametric comparisons between two groups (Frey 2021). For comparisons involving more than two groups, the Kruskal–Wallis test was used (Breslow 1970). These analyses were conducted using the wilcox.test() and kruskal.test() functions in R 4.4.2, ensuring robustness against non-normal distributions.

#### Multicollinearity testing and data preprocessing

Prior to correlation analysis and regression modelling, Variance Inflation Factor (VIF) values were calculated to assess multicollinearity amongst independent variables. Variables with VIF > 10 were considered to exhibit severe multicollinearity and were excluded from subsequent analysis to maintain model validity (Brien and M 2007). This step was performed using the “car” package in R 4.4.2. The 16 retained variables with VIF < 10 are available (Suppl. material 2).

Subsequently, the Shapiro-Wilk test was applied to both dependent and independent variables to assess normality (Shapiro and Wilk 1965). For dependent variables that significantly deviated from a normal distribution (p < 0.05), Box–Cox transformations were performed using the boxcox() function from the “MASS” package in R 4.4.2 to improve distributional properties (Osborne 2010).

#### Correlation and impact analysis between independent variables and bird communities

Following data preprocessing, Spearman's rank correlation coefficient was used to assess monotonic relationships between each independent variable and bird community metrics, including BH', BS, BJ' and BA (Hauke and Kossowski 2011). This analysis was conducted with the “stats” package in R 4.4.2.

To further investigate the combined effects of multiple independent variables on bird community characteristics, a stepwise multiple regression analysis was performed, based on the corrected Akaike Information Criterion (AICc) (Burnham et al. 2011, Roustaei 2024). Building on the Spearman correlation results, the stepwise procedure iteratively adds or removes predictors to identify the optimal subset of variables explaining the variation in response variables. Models with ΔAICc ≤ 2 were considered competitive and, amongst these, the model with the highest Akaike weight (wi) was selected as the best-fit model (Moreira et al. 2015). This modelling was conducted using the “MuMIn” package in R 4.4.2.

## Results

### Summary of bird characteristics

A total of 56 bird species, belonging to seven orders, 28 families and 45 genera, were recorded in the 33 residential areas, with a cumulative individual count of 11,100. Based on individual abundance, the top five most frequently observed species were: the Eurasian Tree Sparrow (*Passer montanus*, 2,476 individuals), the Swinhoe's White-eye (*Zosterops simplex*, 2,077), the Light-vented Bulbul (*Pycnonotus sinensis*, 1,539), the Chinese Blackbird (*Turdus mandarinus*, 1,450) and the Spotted Dove (*Spilopelia chinensis*, 513).

**Figure 3.**
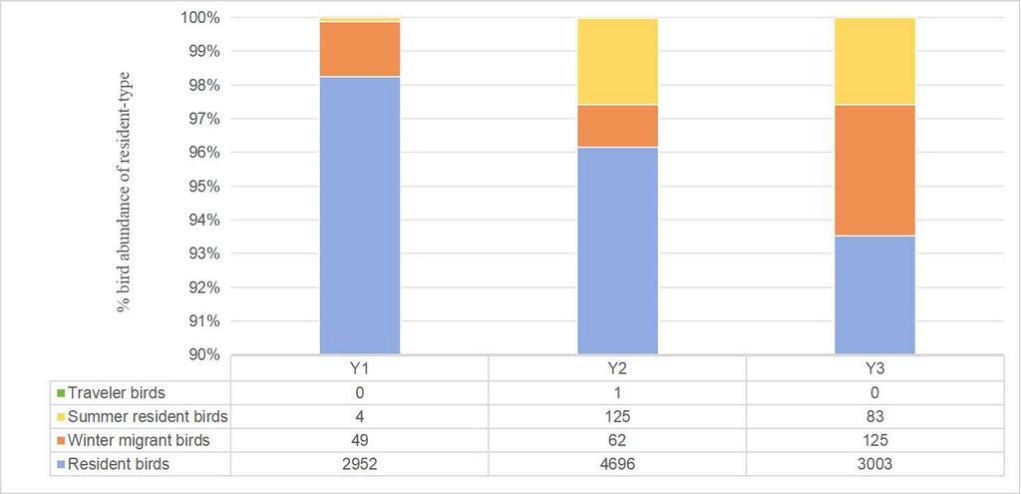
Percentage stacked bar chart illustrating the proportion of resident-type bird abundance across three types of residential areas.

In terms of residency status (Fig. 3), the bird communities across the 33 residential areas were predominantly composed of resident birds and winter migrant birds. Resident species accounted for 40 species, representing 71.43% of the total recorded species, while winter migrant birds comprised 12 species. Summer resident birds and traveller birds were less common, with only three summer resident birds and one traveller bird observed. Across the different residential types, Y3 had significantly more winter migrant birds compared to Y1 and Y2. Both Y2 and Y3 areas also recorded more summer resident birds than Y1, with Y2 exhibiting the highest number of summer species.

**Figure 4.**
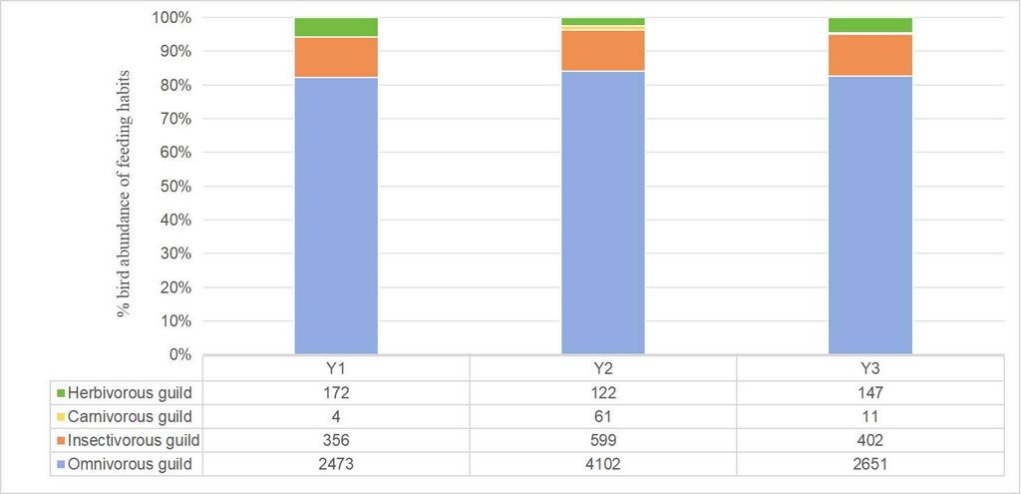
Percentage stacked bar chart illustrating the proportion of bird species' feeding habits across three types of residential areas.

Regarding dietary guilds (Fig. 4), omnivorous birds were the most frequently observed, with 26 species accounting for 47.27% of the total recorded species. Insectivorous guilds, with 19 species, followed this. Carnivorous and herbivorous guilds were the least represented, each with five species recorded. In terms of spatial distribution, Y2 supported a significantly higher number of both carnivorous and omnivorous species compared to Y1 and Y3, whereas Y1 exhibited the highest number of herbivorous species.

### α-Diversity characteristics of residential areas

**Figure 5.**
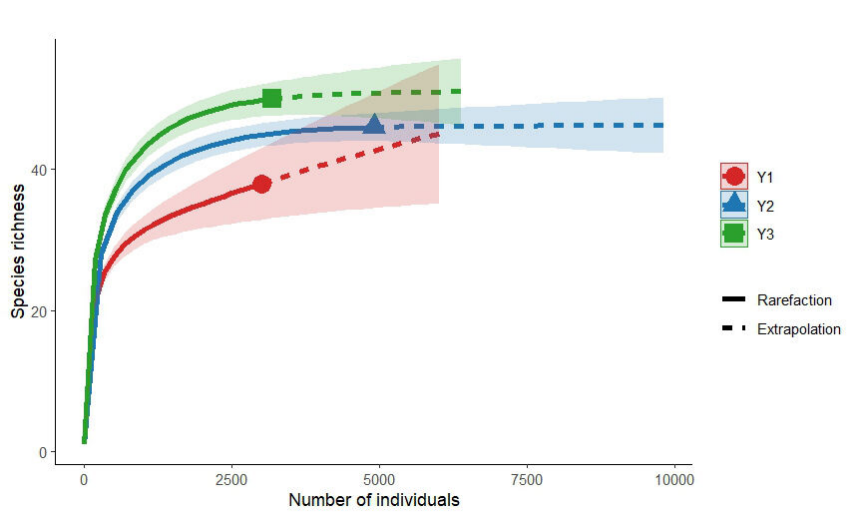
Species accumulation curve of birds in the residential area.

According to the species accumulation curve (Fig. 5), the overall species richness tends to plateau with increasing sample size, indicating that the bird survey data is sufficiently comprehensive and suitable for subsequent analysis.

**Figure 6.**
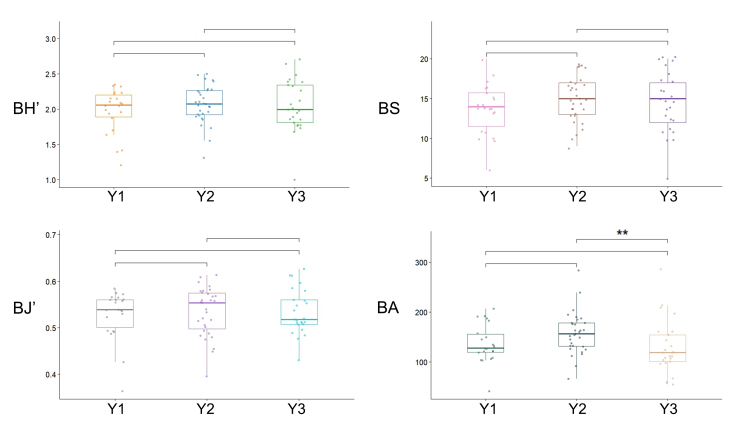
Comparison of bird α-diversity indices across transects in different residential areas.

Notable differences in bird α-diversity were observed amongst the different types of residential areas. Y2 exhibited higher values in the BH', BJ' and BA compared to Y1 and Y3, following the trend Y2 > Y1 > Y3. In terms of BS, however, the pattern was Y2 = Y3 > Y1 (Fig. 6).

The Kruskal–Wallis test revealed no significant overall differences in BH', BS or BJ' amongst the three residential types. However, for BA, a significant difference was observed (P < 0.05). Subsequent pairwise Mann–Whitney U tests revealed that this difference was specifically between the Y2 and Y3 neighbourhoods (P < 0.05).

### Correlation between environmental factors and bird α-Diversity in residential areas

**Figure 7.**
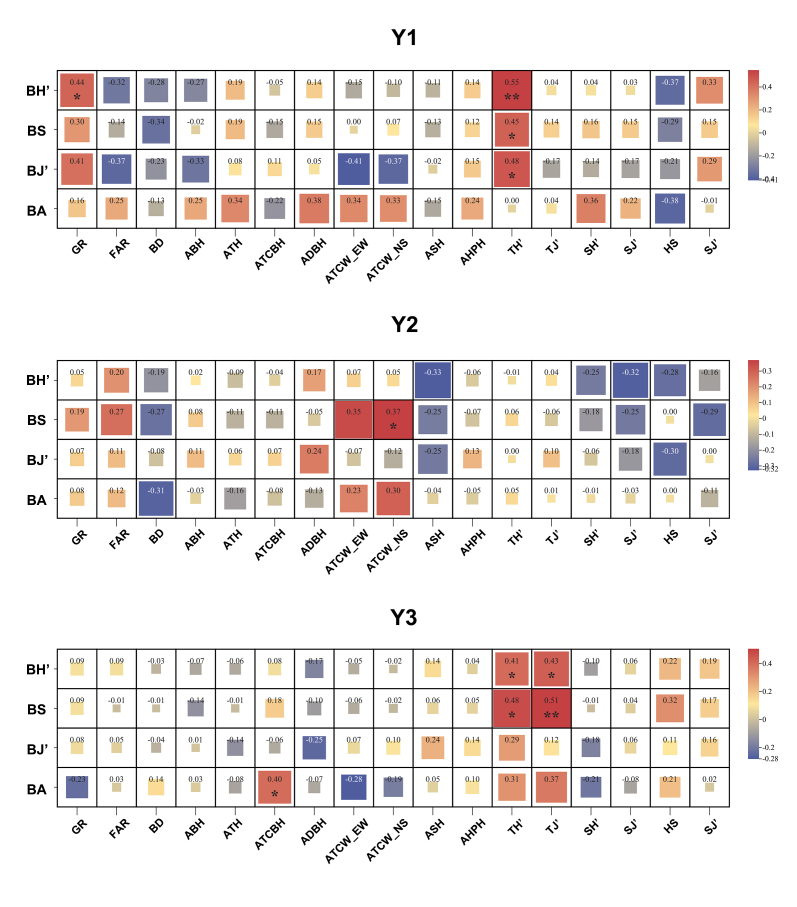
Heatmap of correlations between residential environmental factors and bird α-diversity metrics.

Spearman correlation analysis (Fig. 7) revealed distinct patterns across residential area types (Suppl. material 2). In Y1 areas, bird diversity showed a significant positive correlation with GR and a highly significant positive correlation with TH'. Both BS and BJ' were also significantly positively correlated with TH'. In Y2 areas, BS was significantly positively correlated with the ATCW_NS. In Y3 areas, both BH' and BS were significantly positively correlated with TH'. BH' also showed a significant positive correlation with TJ', while BS was highly significantly correlated with TJ'. Additionally, BA showed a significant positive correlation with the ATCBH.

### Regression analysis between environmental variables and bird α-Diversity in residential areas

The results of the stepwise regression analysis (Table 1) reveal variations in the combinations of significant environmental predictors across different residential types. In Y1 areas, BH' was negatively associated with BD and FAR and positively associated with ATH and TH' (R² = 0.722). BS was negatively correlated with BD and positively correlated with ATH and TH' (R² = 0.443). The model for BJ' included HJ', ASH and TJ' as predictors (R² = 0.718). The model for BA identified GR, AHPH and TJ' as positive predictors (R² = 0.620). In Y2 areas, BH' was negatively associated with HS, SJ' and BD (R² = 0.270). The model for BS included ATCW_EW and FAR, both of which were positively associated (R² = 0.359). The model for BJ' retained only HS as a predictor (R² = 0.070). In the model for BA, TJ' was a positive predictor, while BD, ABH and ATH were negatively associated (R² = 0.715). In Y3 areas, TH' was the sole predictor of BH' (R² = 0.195). The BS model included TJ' and TH', both of which had positive effects (R² = 0.318). The model for BJ' included only TH' (R² = 0.071). For BA, the model included SH' and ASH as negative predictors, while ATCBH, TJ' and TH' were positively associated (R² = 0.562).

## Discussion

### Differences in bird community characteristics amongst residential types

Between 1 October 2023 and 30 September 2024, 12 bird surveys were conducted across 33 residential areas along the Min River in Fuzhou. A total of 56 bird species were recorded, spanning seven orders, 28 families and 45 genera. Resident birds dominated the assemblage, accounting for 40 species, suggesting that residential environments in Fuzhou provide relatively stable food resources and suitable habitats for year-round habitation.

Interestingly, the number of winter migrant birds was significantly higher in newly-developed Y3 areas compared to the other residential types, which may be attributed to more diverse habitats and abundant food resources in Y3 (Jokimäki and Suhonen 1998), particularly the prevalence of winter-fruiting ornamental plants such as mandarin orange (*Citrus reticulata*), lemon (*Citrus limon*) and bitter orange (Citrus × aurantium), which are commonly planted in newer developments.

Dietary guild analysis showed that omnivorous species had the highest species richness and abundance across all residential types, followed by insectivorous birds. This pattern aligns with findings from urban bird surveys in other subtropical cities, such as Guangzhou (Wu et al. 2024). The dominance of omnivores reflects their broad dietary plasticity, which enables them to exploit a wide range of food sources, including anthropogenic waste and cultivated horticultural plants, thereby enhancing their adaptability to dynamic urban environments. Their capacity to cope with seasonal and spatial fluctuations in resource availability also likely contributes to their ecological success in cities (Jokimäki and Suhonen 1998, Marcacci et al. 2021, Ding et al. 2025).

Notably, Y2 areas supported significantly higher numbers of both omnivorous and carnivorous birds compared to Y1 and Y3, which may be linked to the presence of naturalistic waterbodies within Y2’s cluster green spaces, which attract carnivorous species, such as the Little Egret (*Egretta garzetta*), Chinese Pond Heron (*Ardeola bacchus*) and Black-crowned Night Heron (*Nycticorax nycticorax*).

Analysis of bird diversity indices revealed that Y2 neighbourhoods exhibited the highest levels of bird diversity, evenness and abundance, indicating more complex community structures and more balanced species distributions. This finding is consistent with the study by Rebecca Thompson et al. (2022), who reported that parks of intermediate age (40–60 years) had significantly greater overall biodiversity compared to both older central parks (> 60 years) and younger suburban parks (< 40 years). The only exception was species richness, which showed no significant difference between intermediate and older central parks, although both were significantly higher than those in younger parks (Thompson et al. 2022).

These trends may be picking up on what has been indicated elsewhere that intermediate urbanisation can lead to higher species richness (Faeth et al. 2011, Filloy et al. 2019). Notably, unlike the findings of Thompson et al. (2022), this study observed comparable bird species richness between the Y2 and Y3 neighbourhoods, which suggests that some newly-developed communities have begun to achieve tangible biodiversity gains through early integration of ecological design principles. This conclusion also supports the view of Aronson et al. (2017) that, even in densely built urban environments, maintaining high biodiversity is possible by incorporating diverse vegetation structures, effective disturbance management and ecological connectivity (e.g. corridors and buffer zones) at the early planning stages (Aronson et al. 2017). Furthermore, Wilcoxon test results indicated a significant difference in bird abundance between Y2 and Y3. While the two types exhibited similar levels of species richness, abundance was lower in Y3 — likely due to higher levels of anthropogenic disturbance, including ongoing construction and increased human activity. In contrast, Y2 areas, having undergone longer ecological establishment and habitat stabilization, were better able to support greater bird abundance.

### Key environmental drivers influencing bird communities

Compared to urban parks, residential green spaces generally support lower bird diversity due to their smaller size and greater spatial isolation (Sorte et al. 2020, Zheng et al. 2025). This pattern aligns with the species–area relationship theory (Lomolino 2000), which posits that smaller habitat patches tend to support fewer species. Nevertheless, these spatially constrained green spaces can still offer considerable ecological value.

During migratory seasons in particular, species such as the Daurian Redstart (*Phoenicurus auroreus*), Stejneger's Stonechat (*Saxicola stejnegeri*) and Barn Swallow (*Hirundo rustica*) are frequently recorded in residential areas, underscoring their role as ecological “stepping stones” within the urban landscape (Partridge and Clark 2018, Buron et al. 2022). Furthermore, these habitats occasionally provide refuge for rare species, including the nationally protected (Class II) Chinese Hwamei (*Garrulax canorus*), highlighting their significance for urban biodiversity conservation.

The ability of residential green spaces to sustain bird diversity largely depends on their specific environmental characteristics, particularly vegetation structure and composition. Spearman’s rank correlation analysis identified distinct patterns in bird responses to vegetation-related variables across residential areas of different construction periods. In the older Y1 areas, bird α-diversity exhibited a significant positive correlation with both greening rate and tree Shannon Index, while richness and evenness were particularly associated with tree Shannon Index. A diverse tree assemblage offers varied food resources and structural niches, as differences in average tree height, average tree crown base height and average tree crown width fulfil the ecological requirements of a wide range of bird species (Fontana et al. 2011).

Previous studies have similarly shown that the vertical stratification provided by mature trees supports territorial or strata-specialist species, thereby contributing to community stability (Vesk et al. 2008, Shanahan et al. 2011). In Y2 areas, bird richness was significantly correlated only with the average tree crown width (N–S). This structural attribute may enhance the functional value of trees as bird habitats by improving shade provision, moderating microclimates and promoting spatial continuity across fragmented green spaces (Fernandez-Juricic and Jokimäki 2001).

In the more recently developed Y3 areas, both bird Shannon Index and richness showed strong positive correlations with tree Shannon Index and tree evenness, whereas bird abundance was positively associated with average tree crown base height. Higher tree evenness likely indicates a more balanced spatial arrangement of tree species, which may support more equitable foraging and nesting opportunities (Kang et al. 2015). Additionally, average tree crown base height directly influences the vertical movement space and concealment available to birds, a factor particularly important for species that forage in the mid- to upper-canopy layers, such as those from the Paridae and Muscicapidae families (Xu et al. 2022b).

### Differences in response mechanisms of key factors across residential areas of different construction periods

In urban ecosystems, bird communities exhibit highly dynamic responses to environmental factors, which are continuously reshaped by changes in urban spatial structure, vegetation composition and the built environment (Clergeau et al. 2001, Marzluff 2017). Furthermore, ecological succession, associated with the temporal progression of urban development, exerts a long-term influence on habitat suitability and species composition. Residential areas constructed during different periods reflect varying planning philosophies, greening strategies and spatial configurations, resulting in heterogeneity in bird sensitivity and response mechanisms to environmental variables.

These differences are further supported by stepwise regression analysis, which identifies divergent combinations of key influencing factors across the three construction periods. In Y1 areas, the regression models include the greatest number of explanatory variables and exhibit relatively high coefficients of determination (R²), suggesting a complex interplay between bird communities and environmental factors. Bird Shannon Index is positively associated with average tree height and tree Shannon Index, but negatively correlated with building density and floor area ratio. These findings are consistent with previous studies, which have shown that high-density built environments limit bird diversity (Amaya-Espinel et al. 2019).

In contrast, structurally and compositionally diverse tree vegetation supports richer bird communities by providing abundant food resources and nesting sites. Bird richness is similarly influenced by average tree height and tree Shannon Index, highlighting the role of vertical vegetation complexity in enhancing habitat quality. Bird Pielou evenness is affected by herbaceous evenness, average herbaceous plant height and tree evenness, indicating that a multilayered vegetation structure can reduce dominance by a few species and promote a more balanced bird community. Although greening rate and average herbaceous plant height were identified as positive predictors of bird abundance in the models, field observations revealed that certain older neighbourhoods with relatively high greening rate suffered from low plant species diversity, poor maintenance and delayed vegetation renewal. These limitations reduce the ecological functionality of the green space. Thus, greening rate alone is not a reliable indicator of habitat quality; more attention should be directed towards introducing native species, enhancing structural complexity and promoting ecological heterogeneity (Catalano 2024).

In Y2 areas, the overall explanatory power of the regression models is relatively low, with only the bird abundance model demonstrating strong predictive capability. This may indicate that bird responses in mid-aged residential settings are more localised or tend to stabilise due to moderate habitat maturation. Bird Shannon Index is negatively correlated with herbaceous species richness, shrub evenness and building density, suggesting that, in the absence of strong vertical structural support, increased herbaceous species richness alone is insufficient to support diverse bird communities (Nielsen et al. 2014). In addition, average tree crown width (N–S) and floor area ratio show positive associations with bird richness, implying that, under moderate development intensity, expanded canopy coverage and balanced vertical development can enhance habitat utilisation efficiency (Amaya-Espinel et al. 2019). The Bird Pielou evenness model includes only herbaceous species richness, with limited explanatory power — possibly reflecting a dominance by a few generalist species, which vegetation metrics cannot fully explain. For bird abundance, tree evenness acts as a positive factor, while building density, average building height and average tree height act as negative predictors. These results underscore that a well-distributed tree canopy supports higher bird abundance. In contrast, densely built, high-rise environments constrain available habitat space, particularly affecting ground-foraging or shrub-dwelling species (Xu et al. 2022).

In Y3 areas, the number of explanatory variables in the models decreases, yet the explanatory power remains moderate. The Bird Shannon Index model retains only tree richness as a significant predictor, indicating that, in these newer developments, the richness and even spatial distribution of introduced tree species are central to supporting bird diversity. Bird richness is positively correlated with both tree Shannon Index and tree richness, re-affirming that increasing species heterogeneity remains a key strategy for attracting a wider range of bird species. Similarly, the Bird Pielou evenness model includes only tree richness, suggesting that bird communities in these areas are in early successional stages and have not yet established complex interspecific competitive dynamics (Blair 1999, McKinney 2006, Shochat et al. 2006). The bird abundance model includes negative predictors, such as average shrub height and shrub Shannon Index. In contrast, the positive predictors are tree-related variables, including tree Shannon Index, tree evenness and average tree crown base height. These findings highlight the importance of optimising vertical vegetation structure. Excessively tall shrubs may obstruct bird movement corridors and overly diverse shrub layers may introduce structural clutter, hindering foraging efficiency and vigilance behaviour for some species (Robinson and Holmes 1984). Conversely, the openness and vertical layering provided by well-structured tree canopies create favourable conditions for a diverse bird assemblage, thereby enhancing local abundance and activity frequency.

## Conclusions

This study examined bird communities across 33 residential neighbourhoods along the Min River in Fuzhou and found that development history significantly influenced avian diversity. Mid-aged neighbourhoods (Y2) supported the highest diversity and abundance, while newly-developed areas (Y3) showed comparable richness, but lower abundance, likely due to disturbance and human activity. Tree diversity and evenness consistently promoted higher bird diversity, whereas building density and floor area ratio had negative effects.

To translate these findings into practice, new neighbourhoods should reserve sufficient green space and prioritise the introduction of diverse and evenly distributed tree species, combined with multi-layered, native vegetation structures. Existing communities can be enhanced through ecological retrofits, such as rooftop greening, rain gardens and the replacement of exotic ornamentals with native species. At the policy level, bird-friendly design guidelines could be incorporated into urban planning approvals to ensure broader and more consistent implementation.

Future research should also address socio-economic factors during urbanisation, such as population density, income level and management practices, which may indirectly shape bird communities. In addition, long-term and seasonal monitoring is needed to capture temporal dynamics and better understand how bird communities respond to ongoing urban development. Such knowledge will help develop more comprehensive strategies for enhancing biodiversity and ecological resilience in residential areas.

## Supplementary Material

E92ABE78-52BB-50D1-AEF1-8FF3191EE187 10.3897/BDJ.13.e171169.suppl1 Supplementary material 1 One-year bird survey data Data type Abundance Brief description One-year bird survey data of 33 residential areas along the Min River in Fuzhou, China. File: oo_1407686.xlsx https://binary.pensoft.net/file/1407686 Ding Yinghong

554017D9-7FA5-5BAE-9411-BC24946206AB 10.3897/BDJ.13.e171169.suppl2 Supplementary material 2 Values of all independent variables Data type Value File: oo_1407688.xlsx https://binary.pensoft.net/file/1407688 Ding Yinghong, Pan Deng, Wang Yuhan

## Figures and Tables

**Table 1.** AICc-based summary of optimal regression models linking residential environmental factors and avian α-Diversity.

**Response Variable**	**Residential area type**	**Model**	**R**²	**F**	**AICc**	**wi**	**SE**
BH'	Y1	Shannon = 1.230 - 0.013BD + 0.051ATH + 0.451TH' - 0.111FAR	0.722	14.62	-4	0.281	BD (*), FAR (*), ATH (**), TH' (***)
	Y2	Shannon = 4.417 - 0.043HS - 2.426SJ' - 0.021BD	0.270	4.707	5.2	0.295	BD(**)
	Y3	Shannon = 1.296 + 0.390TH'	0.195	6.826	21.1	0.370	TH'(*)
BS	Y1	Richness = -1.552 - 0.402BD + 1.822ATH + 12.717TH'	0.443	6.575	159.8	0.398	BD(*), ATH(**), TH'(**)
	Y2	Richness = 12.448 - 13.362SH' 3.068ATCW_EW + 10.081FAR	0.359	6.602	221.2	0.293	ATCW_EW(**), FAR(**)
	Y3	Richness = −63.429 + 119.883TJ' + 10.804TH'	0.318	6.587	194.4	0.283	TJ'(*), TH'(*)
BJ'	Y1	Pielou = -0.358 + 0.345HJ'- 0.001BD - 0.012ATCW_EW - 0.029ASH - 0.176TJ'	0.718	11.69	-111.8	0.241	BD(*), ASH(*), ATCW_EW(*), TJ'(*), HJ'(***)
	Y2	Pielou = -0.332 - 0.004HS	0.070	3.253	-133.1	0.179	-
	Y3	Pielou = -0.393 + 0.019TH'	0.071	2.842	-108.2	0.303	-
BA	Y1	Abundance = -46.520 + 65.321GR + 3.921AHPH + 1.783ATCW_EW - 6.932ASH + 66.936TJ' + 4.204FAR	0.620	6.716	133.4	0.271	ASH(*), GR(**), AHPH(**), ATCW_EW(**), TJ'(**), FAR(**)
	Y2	Abundance = 55.240 - 0.737BD - 33.794GR - 0.242ABH + 2.344ATCW_NS - 1.147ATH - 0.439ATCBH + 23.472TJ'	0.715	11.74	169.4	0.255	TJ'(*), BD(**), GR(**), ABH(**), ATCW_NS(**), ATH(**), ATCBH(**)
	Y3	Abundance = −4.319 − 15.291SH' − 19.054ASH + 3.333ATCBH + 76.218TJ' + 5.340TH'	0.562	7.153	162.1	0.227	SH'(*), ASH(*), ATCBH(*), TJ'(*), TH'(*)
